# The additive effect of essential hypertension on coronary artery plaques in type 2 diabetes mellitus patients: a coronary computed tomography angiography study

**DOI:** 10.1186/s12933-021-01438-9

**Published:** 2022-01-04

**Authors:** Yu Jiang, Yuan Li, Ke Shi, Jin Wang, Wen-Lei Qian, Wei-Feng Yan, Tong Pang, Zhi-Gang Yang

**Affiliations:** Department of Radiology, West China Hospital, Sichuan University, 37# Guo Xue Xiang, Chengdu, 610041 Sichuan China

**Keywords:** Hypertension, Coronary artery plaque, Diabetes, Coronary computed tomography angiography, Coronary artery disease

## Abstract

**Background:**

The effect of comorbid hypertension and type 2 diabetes mellitus (T2DM) on coronary artery plaques examined by coronary computed tomography angiography (CCTA) is not fully understood. We aimed to comprehensively assess whether comorbid hypertension and T2DM influence coronary artery plaques using CCTA.

**Materials and methods:**

A total of 1100 T2DM patients, namely, 277 normotensive [T2DM(HTN−)] and 823 hypertensive [T2DM(HTN +)] individuals, and 1048 normotensive patients without T2DM (control group) who had coronary plaques detected on CCTA were retrospectively enrolled. Plaque type, coronary stenosis, diseased vessels, the segment involvement score (SIS) and the segment stenosis score (SSS) based on CCTA data were evaluated and compared among the groups.

**Results:**

Compared with patients in the control group, the patients in the T2DM(HTN−) and T2DM(HTN +) groups had more partially calcified plaques, noncalcified plaques, segments with obstructive stenosis, and diseased vessels, and a higher SIS and SSS (all P values < 0.001). Compared with the control group, T2DM(HTN +) patients had increased odds of having any calcified and any noncalcified plaque [odds ratio (OR) = 1.669 and 1.278, respectively; both P values < 0.001]; both the T2DM(HTN-) and T2DM(HTN +) groups had increased odds of having any partially calcified plaque (OR = 1.514 and 2.323; P = 0.005 and P < 0.001, respectively), obstructive coronary artery disease (CAD) (OR = 1.629 and 1.992; P = 0.001 and P < 0.001, respectively), multivessel disease (OR = 1.892 and 3.372; both P-values < 0.001), an SIS > 3 (OR = 2.233 and 3.769; both P values < 0.001) and an SSS > 5 (OR = 2.057 and 3.580; both P values < 0.001). Compared to T2DM(HTN−) patients, T2DM(HTN +) patients had an increased risk of any partially calcified plaque (OR = 1.561; P = 0.005), multivessel disease (OR = 1.867; P < 0.001), an SIS > 3 (OR = 1.647; P = 0.001) and an SSS > 5 (OR = 1.625; P = 0.001).

**Conclusion:**

T2DM is related to the presence of partially calcified plaques, obstructive CAD, and more extensive coronary artery plaques. Comorbid hypertension and diabetes further increase the risk of partially calcified plaques, and more extensive coronary artery plaques.

## Introduction

Type 2 diabetes mellitus (T2DM) and essential hypertension, two of the most common chronic diseases threatening global public health, are frequently comorbid [1]. Approximately two-thirds of T2DM patients have concomitant hypertension, and the prevalence of hypertension among individuals with diabetes is twice as high as that among nondiabetic patients [1–3]. Both diabetes and hypertension have an extremely detrimental effect on arterial stiffness, and the concurrent presence of these two conditions increases the morbidity and mortality associated with cardiovascular disease due to an adverse positive feedback cycle that exists between them [2, 4].

Coronary artery disease (CAD) is the most common cardiovascular disease. Coronary computed tomography angiography (CCTA) has been widely accepted as a promising non-invasive tool for assessing coronary atherosclerosis. Clinical decision-making and planning for patients based on CCTA data show high agreement with those based on conventional coronary angiography data [5]. Moreover, quantitative global plaque characteristics assessed by CCTA have been reported to predict cardiac death in long-term follow-up [6].

Evidence from previous studies suggests a severe coronary plaque burden in patients with hypertension or T2DM [7–10], whereas the additive effect of essential hypertension complicated with T2DM on coronary plaques assessed by CCTA has rarely been reported. Thus, the use of CCTA for CAD assessment in patients with diabetes and hypertension is necessary. Accordingly, the aim of this study was to explore the effects of comorbid hypertension and T2DM on the type and extent of coronary artery plaques and the coronary artery stenosis caused by these plaques by using CCTA.

## Materials and methods

This study was approved by the Biomedical Research Ethics Committee of our hospital, and written informed consent was waived due to the retrospective nature of this study.

### Study population

Between January 2018 and March 2021, a total of 1420 T2DM patients with coronary plaque detected on CCTA in our hospital were retrospectively reviewed. Coronary plaque was defined as structures > 1 mm^2^ adjacent to the coronary artery lumen or within the vessel lumen that could be distinguished from the surrounding pericardial tissue or the artery lumen itself [11]. The diagnosis of T2DM was made in accordance with the American Diabetes Association guidelines [12]. Hypertension was defined as treatment with antihypertensive drugs or a sustained systolic blood pressure (SBP) ≥ 140 mmHg and/or diastolic blood pressure (DBP) of at least 90 mmHg at rest [4]. The exclusion criteria were as follows: images with significant artefacts or CCTA quality too poor to assess the coronary artery (n = 16); patients with incomplete clinical data (n = 106); patients with a history of coronary artery bypass grafting or stenting (n = 113); patients with concomitant neuroendocrine tumours (n = 42); and patients with severe renal failure [estimated glomerular filtration rate (eGFR) lower than 30 mL/min/1.73 m^2^] (n = 43). Consequently, 1100 T2DM patients with or without essential hypertension [T2DM(HTN +) and T2DM(HTN−), respectively] were enrolled in our study. Another 1048 patients with positive coronary plaque findings based on CCTA but without T2DM or hypertension who were unmatched for age and sex were selected to serve as the control group; the exclusion criteria mentioned above for patients with T2DM also applied to the control subjects.

### CT scanning protocols

The CCTA examinations were performed using Siemens CT scanners (SOMATOM Definition, Siemens Medical Solutions, Forchheim, Germany; and SOMATOM Definition FLASH, Siemens Medical Solutions, Forchheim, Germany) or a Revolution CT scanner (GE Healthcare, Waukesha, WI, USA) with patients in the supine position. An intravenous bolus injection of 70–90 ml (based on body weight) of iodinated contrast agent (iopamidol, 370 mg of iodine/ml; Bracco, Shanghai, China) at a flow rate of 5 ml/s was followed by a 30 ml saline flush at the same flow rate. The scan range was from the tracheal bifurcation to 20 mm below the inferior cardiac apex. For the SOMATOM Definition systems, the parameters were as follows: tube voltage, 100–120 kV; tube current, 220 mAs; collimation, 64/128 × 0.5 mm; and rotation time, 0.33 s–0.4 s. For the Revolution CT, the parameters were as follows: the tube voltage and tube current were modulated automatically by kV Assist and Smart-mA on the basis of the scout image, collimation was 256 × 0.625 mm, and rotation time was 0.28 s. Either a retrospective electrocardiogram-gated or a prospective electrocardiographic gating protocol was used for CCTA image acquisition. Subsequently, the initial data set was reconstructed upon completion of the scan, and images were transferred to image-processing workstations (Syngo-Imaging, Siemens Medical Solution Systems, Forchheim, Germany; or AW VolumeShare5, GE Healthcare, Waukesha, WI, USA) for image analysis.

### CCTA analysis

**Fig. 1:**
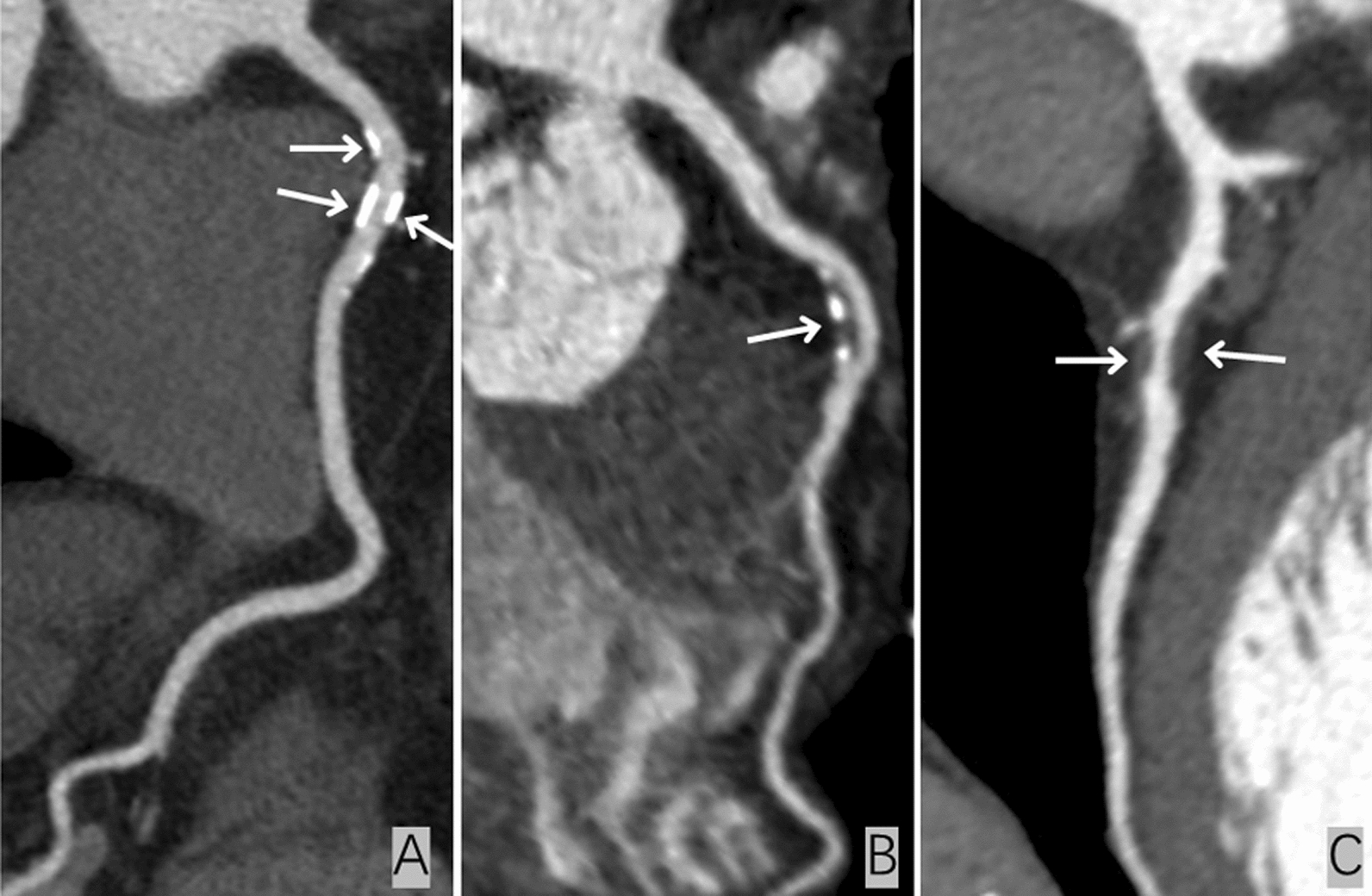
Representative CCTA images of different types of coronary artery plaques. **A** Calcified plaque, **B** partially calcified plaque and **C** noncalcified plaque

**Fig. 2:**
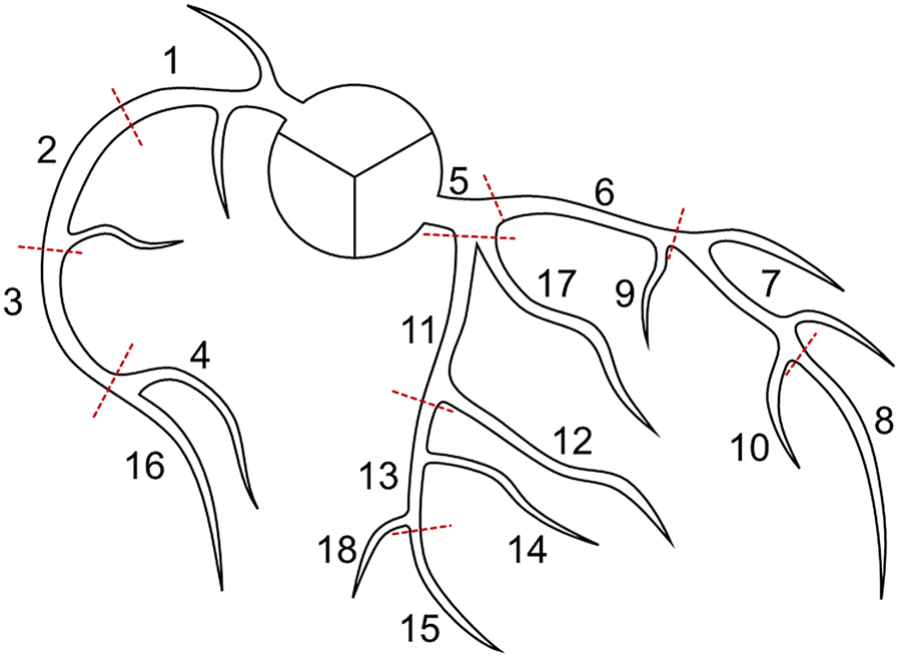
Coronary artery segments: 1 proximal segment of the right coronary artery (RCA); 2 middle segment of the RCA; 3 distal segment of the RCA; 4 right posterior descending artery; 5 left main coronary artery; 6 proximal segment of the left anterior descending artery (LAD); 7 middle segment of the LAD; 8 distal segment of the LAD; 9 first diagonal branch; 10 s diagonal branch; 11 proximal segment of the left circumflex (LCX); 12 first obtuse marginal branch; 13 distal segment of the LCX; 14 s obtuse marginal branch; 15 left posterior descending artery; 16 right posterolateral artery; 17 ramus intermedius branch; 18 left posterolateral branch

The presence of plaque and luminal stenosis was assessed for each evaluable coronary segment. Plaques were visually classified as calcified plaque (CT attenuation of plaque higher than contrast-enhanced coronary lumen), noncalcified plaque (CT density of plaque lower than contrast-enhanced lumen without any calcification) and partially calcified plaque (both calcified and noncalcified components present in a single plaque) (Fig. 1) [13]. The severity of stenosis was quantified and graded by visual estimation using a scale based on the Coronary Artery Disease-Reporting and Data System (CAD-RADS) [14]: grade 0 (absence of plaques), grade 1(< 25% stenosis), grade 2 (25–49% stenosis), grade 3 (50–69% stenosis), grade 4 (70–99% stenosis), or grade 5 (total occlusion). Any presence of stenosis greater than 50% was defined as obstructive stenosis, and nonobstructive CAD presented without any obstructive stenosis. Multivessel disease was defined as ≥ 2 diseased vessels. The segment involvement score (SIS) and segment stenosis score (SSS) were calculated for all patients. The SIS was calculated as the total number of coronary artery segments with plaques. The SSS was calculated as the summation of the stenosis grades of all 18 individual segments according to the Society of Cardiovascular Computed Tomography Guidelines Committee (Fig. 2) [15]. Two cardiovascular radiologists blinded to the clinical information of the patients analysed the images independently. The two observers reached a consensus by discussion when there were disagreements.

### Statistical analysis

Continuous variables are expressed as the mean ± standard deviation and categorical variables are presented as numbers (%) in this study. The comparison of continuous variables among groups was performed using one-way analysis of variance or the Kruskal–Wallis rank test, and comparison of categorical variables was performed using the chi-square test followed by Bonferroni’s post-hoc test. The comparisons for diabetes duration and diabetes treatment between the T2DM(HTN +) and T2DM(HTN−) groups were performed using the Mann–Whitney U test and chi-square test, respectively. Multivariate logistic regression adjusted for confounding factors was used to analyse the associations of plaque characteristics among the groups. The presence of each type of plaque, extent of luminal narrowing, involved branch, SIS and SSS were statistically analysed for each patient. For all analyses, a two-tailed P value of less than 0.05 was considered indicative statistical significance. All statistical analyses were performed using SPSS software (version 24.0; IBM, Armonk, New York, USA).

## Results

### Study population and clinical baseline characteristics

The main clinical characteristics of the participants in the study are summarized in Table 1. A total of 2148 individuals, namely, 277 T2DM(HTN−) patients, 823 T2DM(HTN +) patents, and 1048 patients without T2DM or hypertension were studied. There were 1439 (67.0%) male subjects. The patients in the T2DM(HTN−) group were more likely to be younger. Furthermore, compared with the control group, the patients in the T2DM(HTN-) and T2DM(HTN +) groups were more likely to have higher body mass index (BMI) values, a higher prevalence of dyslipidaemia and statin use, a higher level of fasting blood glucose and plasma triglyceride, and a lower level of high-density lipoprotein cholesterol (HDL-C). Compared with T2DM(HTN−) patients, T2DM(HTN +) patients were more likely to have a lower prevalence of smoking history and use of oral medications for T2DM treatment, a longer duration of T2DM, lower levels of total cholesterol (TC), low-density lipoprotein cholesterol (LDL-C) and eGFR, and higher levels of SBP and DBP readings.

**Table 1:** Baseline characteristics of the study cohort

	Control group (n = 1048)	T2DM(HTN−) (n = 277)	T2DM(HTN +) (n = 823)	P value
Demographics				
Age (years)	70.0 ± 9.3	66.8 ± 10.2^*^	70.3 ± 9.6^§^	< 0.001
Male (%)	707 (67.5%)	197 (71.1%)	535 (65.0%)	0.157
BMI (kg/m^2^)	22.91 ± 3.29	23.97 ± 3.45^*^	24.91 ± 3.24^*§^	< 0.001
Smoking, n (%)	437 (41.7%)	136 (49.1%)	316 (38.4%)^§^	0.007
Dyslipidaemia, n (%)	62 (5.9%)	59 (21.3%)^*^	253 (30.7%)^*§^	< 0.001
Diabetes duration (year)	–	8.4 ± 7.3	9.5 ± 7.7^#^	0.036
Hypertension duration (year)	–	–	12.8 ± 10.9	–
Laboratory data				
HbA1c (%)	–	7.52 ± 1.59	7.49 ± 1.56	0.950
Fasting blood glucose (mmol/L)	5.26 ± 0.73	7.85 ± 3.20^*^	7.57 ± 2.55^*^	< 0.001
Plasma triglycerides (mmol/L)	1.34 ± 0.88	1.60 ± 0.98^*^	1.62 ± 1.10^*^	< 0.001
Total cholesterol (mmol/L)	4.26 ± 1.07	4.12 ± 1.10	3.95 ± 1.13^*^	< 0.001
HDL-C (mmol/L)	1.23 ± 0.41	1.13 ± 0.35^*^	1.12 ± 0.32^*^	< 0.001
LDL-C (mmol/L)	2.52 ± 0.88	2.37 ± 0.91	2.24 ± 0.91^*^	< 0.001
eGFR ((mL/min/1.73 m^2^)	82.74 ± 15.48	84.69 ± 16.72	78.57 ± 18.41^*§^	< 0.001
Haemodynamic variables				
SBP (mmHg)	128 ± 17	128 ± 18	142 ± 19^*§^	< 0.001
DBP (mmHg)	77 ± 11	78 ± 12	81 ± 13^*§^	< 0.001
Diabetes treatment				
Oral, n (%)	–	163 (58.8%)	578 (70.2%)^§^	< 0.001
Insulin, n (%)	–	74 (26.7%)	255 (31%)	0.179
Hypertension treatment				
ACEI/ARB, n (%)	–	–	292 (35.5%)	–
Beta‑blocker, n (%)	–	–	129 (15.7%)	–
Calcium channel blocker, n (%)	–	–	425 (51.6%)	–
Diuretics, n (%)	–	–	66 (8.0%)	–
Lipid-lowering medication				
Statins, n (%)	103 (9.8%)	53 (19.1%)^*^	193 (23.5%)^*^	< 0.001

The values are the mean ± standard deviation or number (%)

*T2DM* type 2 diabetes mellitus, *HTN* hypertension, *BMI* body mass index, *HDL-C* high-density lipoprotein cholesterol, *LDL-C* low-density lipoprotein cholesterol, *eGFR* estimated glomerular filtration rate, *SBP* systolic blood pressure, *DBP* diastolic blood pressure, *ACEI* angiotensin converting enzyme inhibitor, *ARB* angiotensin II receptor blocker

^*^P < 0.017 versus the control group

^§^P < 0.017 versus the T2DM (HTN−) group

^#^P < 0.05 versus the T2DM (HTN−) group

### Comparison of the CCTA findings among the control, T2DM(HTN−) and T2DM(HTN +) groups

**Fig. 3:**
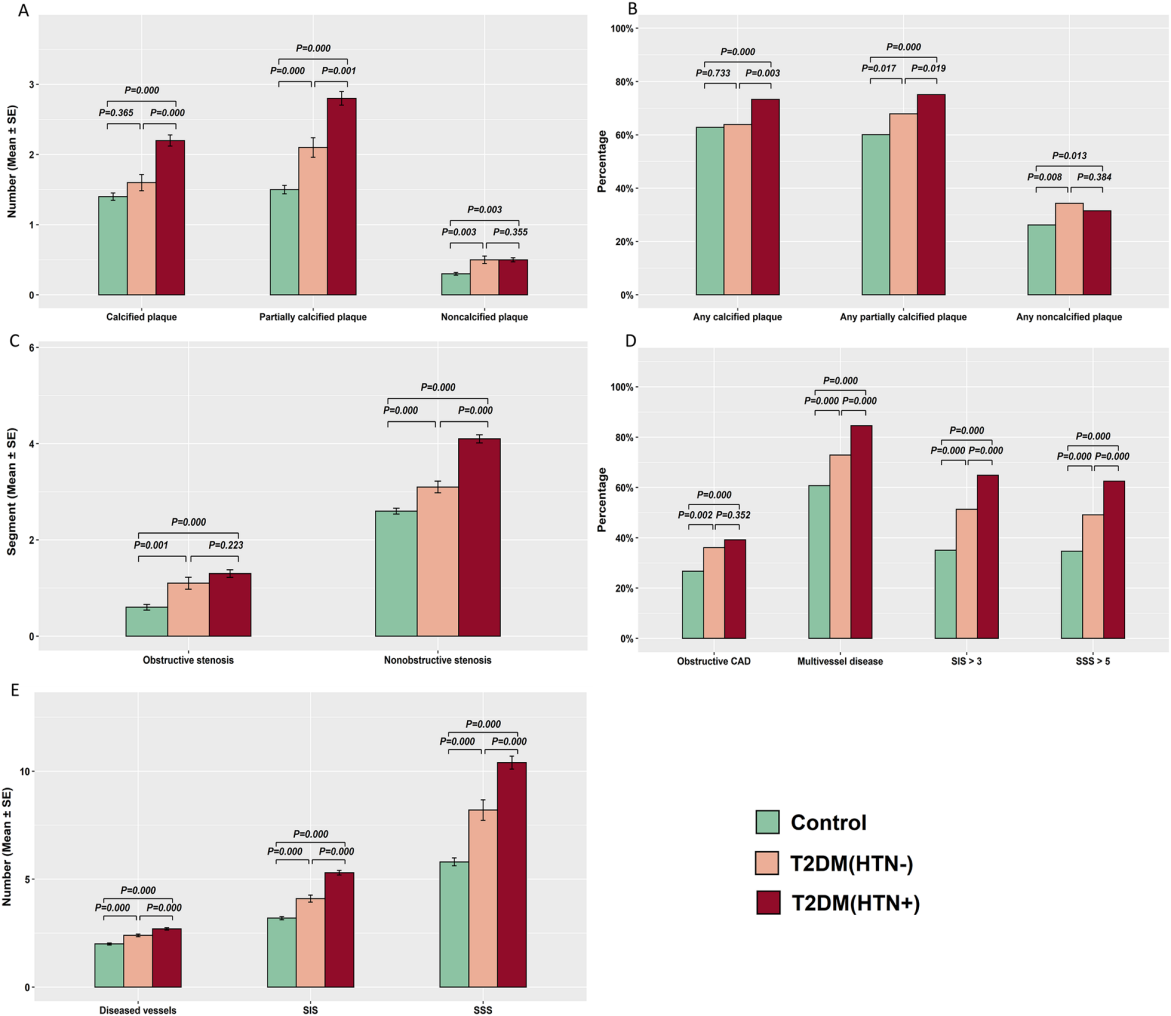
Coronary plaque burden, stenosis and extent of coronary artery plaques detected by coronary computed tomography angiography. The mean value (**A**) and proportion of any presence (**B**) of different plaque types; the mean value of obstructive and nonobstructive coronary artery disease (CAD) (**C**); the proportion of any presence of obstructive CAD, multivessel disease, segment involvement score (SIS) > 3 and segment stenosis score (SSS)  > 5 (**D**); the mean value of diseased vessels, SIS and SSS (E)

A total of 8926 coronary plaques, 5036 diseased vessels and 8868 diseased segments were analysed. The plaque burden, plaque extent and coronary artery stenosis caused by plaques among the groups are shown in Table 2 and Fig. 3. Regarding the types of plaque, the patients in the T2DM(HTN +) group had the largest number of calcified plaques and partially calcified plaques among the three groups [control vs. T2DM(HTN−) vs. T2DM(HTN +), calcified plaques: 1.4 ± 1.7 vs. 1.6 ± 1.9 vs. 2.2 ± 2.3; and partially calcified plaques: 1.5 ± 1.9 vs. 2.1 ± 2.3 vs. 2.8 ± 2.8; P values < 0.001], and the patients in the T2DM(HTN−) group had more partially calcified plaques than those in the control group [2.1 ± 2.3 vs. 1.5 ± 1.9, P < 0.001] (Table 2 and Fig. 3A). The control group had the smallest number of noncalcified plaques [control vs. T2DM(HTN−) vs. T2DM(HTN +): 0.3 ± 0.6 vs. 0.5 ± 0.9 vs. 0.5 ± 0.8, P = 0.001] (Table 2 and Fig. 3A). The T2DM(HTN +) group had a higher proportion of patients with any calcified plaques than the other two groups [control vs. T2DM(HTN−) vs. T2DM(HTN +): 62.8% vs. 63.9% vs. 73.3%, P < 0.001] and a higher proportion of patients with any partially calcified plaques than the control group [control vs. T2DM(HTN +): 60.0% vs. 75.1%, P < 0.001] (Table 2 and Fig. 3B). The patients in the control group had the lowest proportion of noncalcified plaques [control vs. T2DM(HTN−) vs. T2DM(HTN +): 26.2% vs. 34.3% vs. 31.5%, P = 0.007] (Table 2 and Fig. 3B).

**Table 2:** Coronary plaque burden, stenosis and extent of coronary artery plaques detected by CCTA

	Control group (n = 1048)	T2DM(HTN−) (n = 277)	T2DM(HTN +) (n = 823)	P value
Plaque type				
Calcified plaques	1.4 ± 1.7	1.6 ± 1.9	2.2 ± 2.3^*§^	< 0.001
Partially calcified plaques	1.5 ± 1.9	2.1 ± 2.3^*^	2.8 ± 2.8^*§^	< 0.001
Noncalcified plaques	0.3 ± 0.6	0.5 ± 0.9^*^	0.5 ± 0.8^*^	0.001
Stenosis caused by plaques				
Nonobstructive stenosis	2.6 ± 1.9	3.1 ± 2.0^*^	4.1 ± 2.4^*§^	< 0.001
Obstructive stenosis	0.6 ± 1.3	1.1 ± 2.1^*^	1.3 ± 2.3^*^	< 0.001
Diseased vessels	2.0 ± 1.0	2.4 ± 1.1^*^	2.7 ± 1.0^*§^	< 0.001
SIS	3.2 ± 2.3	4.1 ± 2.7^*^	5.3 ± 3.1^*§^	< 0.001
SSS	5.8 ± 5.8	8.2 ± 7.9^*^	10.4 ± 8.7^*§^	< 0.001
Any calcified plaque	658 (62.8%)	177 (63.9%)	603 (73.3%)^*§^	< 0.001
Any partially calcified plaque	629 (60.0%)	188 (67.9%)	618 (75.1%)^*^	< 0.001
Any noncalcified plaque	275 (26.2%)	95 (34.3%)^*^	259 (31.5%)^*^	0.007
Obstructive CAD	280 (26.7%)	100 (36.1%)^*^	323 (39.2%)^*^	< 0.001
Multivessel disease	636 (60.7%)	202 (72.9%)^*^	696 (84.6%)^*§^	< 0.001
SIS > 3	368 (35.1%)	142 (51.3%)^*^	533 (64.8%)^*§^	< 0.001
SSS > 5	364 (34.7%)	136 (49.1%)^*^	514 (62.5%)^*§^	< 0.001

The data are expressed as the mean ± standard deviation or number (%)

*CCTA* coronary computed tomography angiography, *T2DM* type 2 diabetes mellitus, *HTN* hypertension, *SIS* segment involvement score, *SSS* segment stenosis score

^*^P < 0.017 versus the control group

^§^P < 0.017 versus the T2DM (HTN−) group

Regarding segment stenosis caused by plaques, patients in the control group had the fewest segments with obstructive stenosis among the three groups [control vs. T2DM(HTN−) vs. T2DM(HTN +): 0.6 ± 1.3 vs. 1.1 ± 2.1 vs. 1.3 ± 2.3, P < 0.001] (Table 2 and Fig. 3C). There were fewer patients with obstructive CAD detected in the control group than in the T2DM(HTN−) and T2DM(HTN +) groups [control vs. T2DM(HTN−) vs. T2DM(HTN +):26.7% vs. 36.1% vs. 39.2%, P < 0.001] (Table 2 and Fig. 3D).

**Fig. 4:**
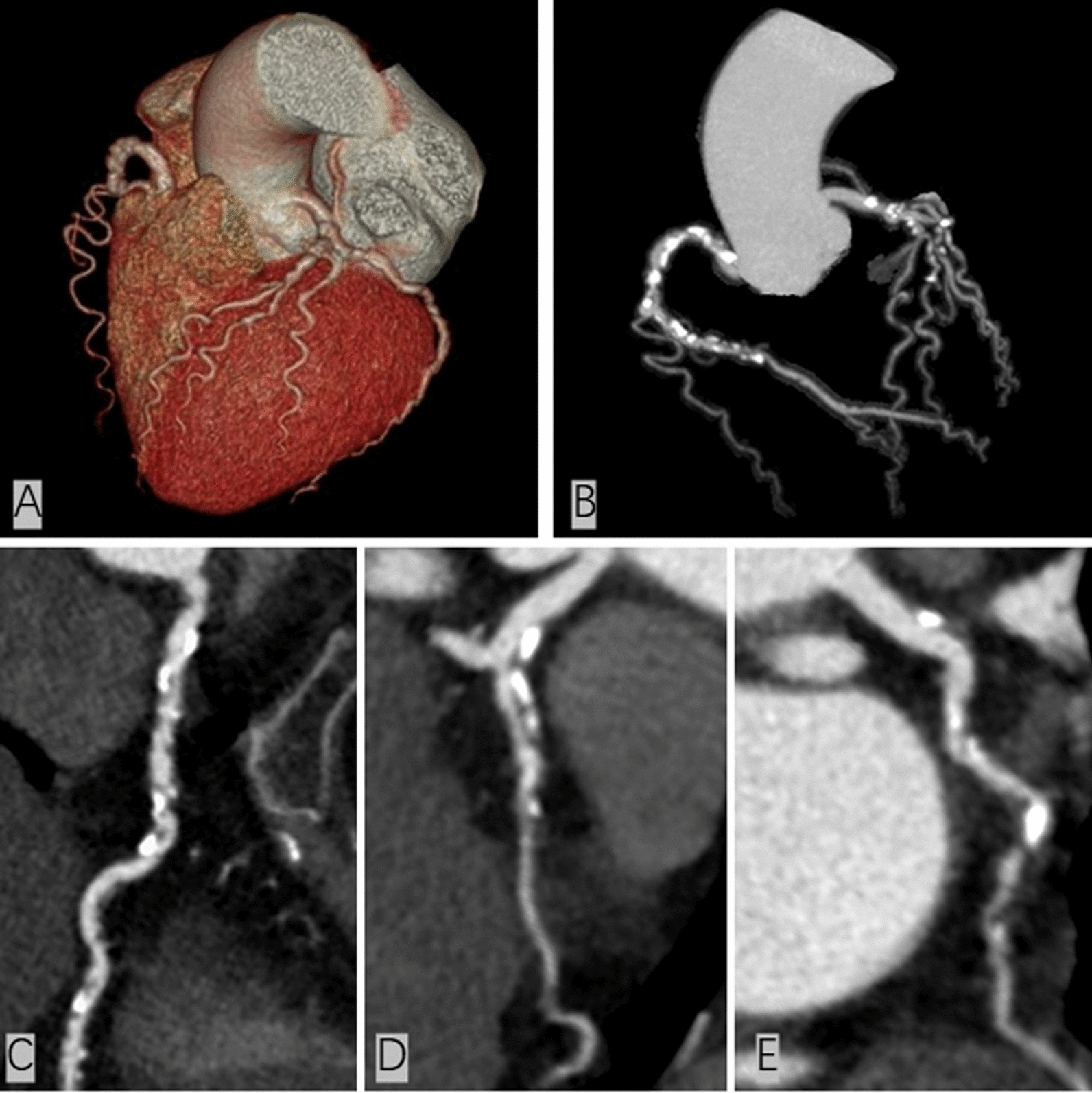
Multivessel disease in a 69-year-old female with type 2 diabetes mellitus and hypertension. Volume rendering image (**A**) and maximum intensity projection (**B**) show the unsmooth edge of coronary arteries with scattered plaques, and curvature plane reconstruction images (**C**–**E**) show the diffuse partially calcified plaques distributed in the coronary arteries

Regarding the extent and severity of plaques, the T2DM(HTN +) group had the greatest number of diseased vessels and segments and the highest SSS, followed by the T2DM(HTN−) group [control vs. T2DM(HTN−) vs. T2DM(HTN +): diseased vessels, 2.0 ± 1.0 vs. 2.4 ± 1.1 vs. 2.7 ± 1.0; SIS, 3.2 ± 2.3 vs. 4.1 ± 2.7 vs. 5.3 ± 3.1; SSS, 5.8 ± 5.8 vs. 8.2 ± 7.9 vs. 10.4 ± 8.7, all P values < 0.001] (Table 2 and Fig. 3E). In addition, among the three groups, the T2DM(HTN +) group had the largest proportion of individuals with multivessel disease (Fig. 4), an SIS > 3 and an SSS > 5, followed by the T2DM(HTN−) group [control vs. T2DM(HTN−) vs. T2DM(HTN +): multivessel disease, 60.7% vs. 72.9% vs. 84.6%; SIS > 3, 35.1% vs. 51.3% vs. 64.8%; SSS > 5, 34.7% vs. 49.1% vs. 62.5%, all P-values < 0.001] (Table 2 and Fig. 3D).

### Multivariate regression analysis of CCTA findings among the control, T2DM(HTN−) and T2DM(HTN +) groups

Multivariate regression analysis was performed to control for age, sex, BMI, smoking history, dyslipidaemia, fasting blood glucose, plasma triglyceride, TC, HDL-C, LDL-C, eGFR, SBP, DBP, and statin use, using the control group as the reference group (Table 3, Model 1). Compared to those in the control group, the patients in the T2DM(HTN +) group had increased odds of having any calcified plaque [odds ratio (OR): 1.669; 95% confidence interval (CI) 1.351–2.062, P < 0.001]. Both the T2DM(HTN−) and T2DM(HTN +) groups had increased odds of having any partially calcified plaque [OR (95% CI) for T2DM(HTN−): 1.513 (1.133–2.022); and for T2DM(HTN +): 2.323 (1.870–2.887), respectively; both P values < 0.001], obstructive CAD [OR (95% CI) for T2DM(HTN−): 1.629 (1.222–2.170), P = 0.001, and for T2DM(HTN +): 1.992 (1.616–2.455), P < 0.002, respectively], multivessel disease [OR (95% CI) for T2DM(HTN−): 1.892 (1.393–2.568), and for T2DM(HTN +): 3.372 (2.619–4.342), respectively; both P values < 0.001], an SIS > 3 [OR (95% CI) for T2DM(HTN−): 2.233 (1.684–2.961), and for T2DM(HTN +): 3.769 (3.046–4.663), respectively; both P values < 0.001] and an SSS > 5 [OR (95% CI) for T2DM(HTN−): 2.057 (1.554–2.722), and for T2DM(HTN +): 3.580 (2.902–4.417), respectively; both P values < 0.001].

**Table 3:** Multivariate regression analysis of the CCTA findings

	Model 1					Model 2		
	Control group (n = 1048)	T2DM(HTN−) (n = 277)		T2DM(HTN +) (n = 823)		T2DM(HTN−) (n = 277)	T2DM(HTN +) (n = 823)	
		OR (95% CI)	P value	OR (95% CI)	P value		OR (95% CI)	P value
	Reference					Reference		
Any calcified plaque		–	0.170	1.669 (1.351–2.062)	< 0.001		–	0.074
Any partially calcified plaque		1.513 (1.133–2.022)	0.005	2.323 (1.870–2.887)	< 0.001		1.561 (1.144–2.130)	0.005
Any noncalcified plaque		–	0.084	1.278 (1.033–1.580)	0.024		–	0.604
Any obstructive CAD		1.629 (1.222–2.170)	0.001	1.992 (1.616–2.455)	< 0.001		–	0.161
Multivessel disease		1.892 (1.393–2.568)	< 0.001	3.372 (2.619–4.342)	< 0.001		1.867 (1.337–2.608)	< 0.001
SIS > 3		2.233 (1.684–2.961)	< 0.001	3.769 (3.046–4.663)	< 0.001		1.647 (1.231–2.204)	0.001
SSS > 5		2.057 (1.554–2.722)	< 0.001	3.580 (2.902–4.417)	< 0.001		1.625 (1.221–2.162)	0.001

### The additive effect of hypertension on the types and extent of coronary artery plaques, and segment stenosis in T2DM patients

As shown in Model 2 (Table 3), multivariate regression analysis was performed, adjusting for the duration of diabetes and use of oral medication for T2DM in addition to the confounding factors in Model 1 (Table 3). Compared to the patients in the T2DM(HTN−) group, those in the T2DM(HTN +) group had an increased risk of any partially calcified plaque [OR (95% CI): 1.561 (1.144–2.130), P = 0.005], multivessel disease [OR (95% CI): 1.867 (1.337–2.608), P < 0.001], an SIS > 3 [OR (95% CI): 1.647 (1.231–2.204), P = 0.001] and an SSS > 5 [OR (95% CI): 1.625 (1.221–2.162), P = 0.001]. There was no significant difference in the presence of calcified plaques, noncalcified plaques or obstructive CAD between the T2DM(HTN−) and T2DM(HTN +) groups (all P values > 0.05).

## Discussion

This study investigated the effects of comorbid hypertension and diabetes on the presence of coronary artery plaques, coronary stenosis, and the extent of coronary plaques. The main findings of the present study were as follows: first, the T2DM patients had more partially calcified plaques than the nondiabetic patients, and the T2DM patients with hypertension had more partially calcified and calcified plaques than the patients with T2DM alone; second, compared with the control group, higher number of the T2DM patients had obstructive CAD; third, the T2DM patients had more extensive and severe CAD and when they also had hypertension, the number of patients with more extensive and severe CAD were even higher; finally, the multivariate analysis further indicated that comorbid hypertension and T2DM increased the risk of any partially calcified plaques, and extensive and severe CAD.

Hypertension is one of the most common comorbidities among diabetes patients, and, according to a previous epidemiological study, occurs in 77.1% and 66.3% of adults with diabetes according to the American College of Cardiology/American Heart Association and American Diabetes Association, respectively [3]. Another study reported that 79.2% of T2DM patients with hypertension had CAD [16]. Similar to previous studies, our data showed that approximately three out of four T2DM patients with CAD also had hypertension. Comorbid T2DM and hypertension seem to result in common arterial wall damage in the form of calcification [17]; however, the combined effect of the two diseases on CAD is not fully understood. Thus, this study was performed to acquire a deeper understanding of the additive effects of comorbid hypertension and T2DM on CAD.

### The additive effect of hypertension on plaque type in T2DM

A previous study indicated that T2DM patients had a higher burden of partially calcified plaques than nondiabetic patients [16], which was also observed in our study. The mechanisms of vascular calcification in diabetes include oxidative stress, mineral metabolism alteration, endothelial dysfunction, and increased inflammatory cytokine production [18, 19]. Regarding the risk of plaque, the presence of diabetes without hypertension tended to confer a higher risk of any partially calcified plaques in our study [T2DM(HTN−) vs. control, OR = 1.513], and the risk was further increased when hypertension was present [T2DM(HTN +) vs. T2DM(HTN−), OR = 1.561]. Hypertension can also promote the progression of atherosclerosis [20]. Through altered forces of wall shear stress and increased oxidative stress, hypertension destroys endothelial function, and then triggers a series of potent pathophysiological processes, including vascular smooth muscle cell proliferation, vascular remodelling, and apoptosis, and increases cellular permeability with an increase in adhesion molecules, which eventually accelerate the development of plaques [20–23].

### Obstructive CAD in T2DM and hypertension

In our data, obstructive CAD was less frequently observed than nonobstructive CAD in all the groups. However, the annual rate of major adverse cardiac events is higher in patients with obstructive CAD than in those with nonobstructive CAD according to a previous study [24]. Our data revealed a higher risk of any obstructive CAD in patients with T2DM than in those without T2DM, while there was no significant difference in the risk of any obstructive CAD between T2DM patients with and without hypertension. Although hypertension did not increase the risk of obstructive CAD on the basis of the presence of T2DM after adjustment for confounding factors in our study, the increased proportion of T2DM patients with obstructive CAD relative to that of patients without T2DM should not be ignored. The presence of coronary artery narrowing caused by plaques in patients with diabetes increases the probability of an acute plaque event [25]. In addition, the prognosis of diabetes with obstructive CAD was found to be worse than that of diabetes with nonobstructive disease over a 5-year follow-up [26].

### Hypertension aggravate extent and severity of CAD in T2DM

According to the present study, T2DM (HTN−) patients had a higher risk of more extensive and severe CAD than patients in control group after adjusting for confounding factors, and T2DM patients with hypertension had a higher risk than those without hypertension. A possible explanation could be the common pathogenesis and development process of atherosclerosis shared by diabetes and CAD, and comorbid diabetes and hypertension dramatically increase the risk of cardiovascular disease [1, 27]. The glycaemic variability in diabetic patients is closely related to oxidative stress and endothelial function; poor glycaemic control in patients with T2DM has an adverse effect on CAD severity, and long-term glucose variability correlates with the risk of myocardial infarction [28–30]. Regarding the aetiology of hypertension, there is a mechanism by which the autonomic nervous system plays a central role in the pathophysiology of hypertension that has also been linked to diabetes [31]. Furthermore, in patients with T2DM and hypertension, a complicated haemodynamic feedback cycle exists, and this greatly increases the severity of cardiovascular diseases [2].

Patients with extensive CAD can benefit from surgical revascularization [32, 33]. For multivessel CAD, coronary artery bypass grafting was correlated with a lower rate of long-term major adverse cardiac or cerebrovascular events relative to percutaneous coronary intervention for both stable ischaemic heart disease and acute coronary syndromes [34]. Previous studies also indicate a mortality benefit of appropriate revascularization in patients with multivessel disease, especially for those with diabetes [35, 36]. Thus, the assessment of the extent of coronary artery plaques in patients with comorbid diabetes and hypertension could be important for proper clinical decision-making.

## Limitation

There are some limitations of this study. First, the evaluation of coronary plaque was based on visual assessment of CCTA images. However, visual assessment of CCTA has been widely used and validated for evaluating the characteristics and progression of coronary plaques in CAD patients [37, 38]. The quantification of coronary plaques in T2DM patients requires further exploration. Second, selection bias is inevitable since this is a single-centre study. Therefore, further multicentre studies should be performed to validate the findings in this study. Third, only comorbid hypertension and diabetes were studied, while individuals with hypertension alone were not enrolled in our study. However, the effects of hypertension alone on CAD have been reported in previous studies [7, 8]. Finally, the grades of coronary stenosis in the CCTA findings of this study were not compared with invasive coronary angiography results, because CCTA for the evaluation of coronary plaques has been widely accepted and can further distinguish different types of plaques.

## Conclusion

In conclusion, T2DM is related to the presence of partially calcified plaques, obstructive CAD and more extensive coronary artery plaques. Comorbid hypertension and diabetes further increase the risk of partially calcified plaques and more extensive coronary artery plaques; however, they have little effect on the risk of obstructive CAD compared with T2DM without hypertension.

## Data Availability

The datasets used and analysed during the current study are available from the corresponding author on reasonable request.

## Abbreviations

BMI: Body mass index
CAD: Coronary artery disease
CCTA: Coronary computed tomography angiography
CI: Confidence interval
DBP: Diastolic blood pressure
eGFR: Estimated glomerular filtration rate
HDL-C: High-density lipoprotein cholesterol
LDL-C: Low-density lipoprotein cholesterol
OR: Odds ratio
SBP: Systolic blood pressure
SIS: Segment involvement score
SSS: Segment stenosis score
T2DM: Type 2 diabetes mellitus
TC: Total cholesterol
